# Correlation of long non-coding RNA expression with metastasis, drug resistance and clinical outcome in cancer

**DOI:** 10.18632/oncotarget.2469

**Published:** 2014-09-29

**Authors:** Ehsan Malek, Sajjeev Jagannathan, James J. Driscoll

**Affiliations:** ^1^ The Vontz Center for Molecular Studies, University of Cincinnati College of Medicine, Cincinnati, OH; ^2^ Division of Hematology and Oncology, University of Cincinnati College of Medicine, Cincinnati, OH; ^3^ Department of Cancer Biology, University of Cincinnati College of Medicine, Cincinnati, OH

**Keywords:** non-coding RNAs, long-non-coding RNAs, microRNAs, *HOTAIR*, overall survival

## Abstract

The therapeutic response and clinical outcome of patients diagnosed with the same cancer type and that receive identical treatment is highly variable to reflect the genetic heterogeneity within tumor cells. Non-coding RNAs (ncRNAs) are recently discovered molecules that regulate eukaryotic gene expression and represent a significant advance towards a better understanding of the mechanisms that govern cellular growth. NcRNAs are essential for the proper regulation of cell proliferation and survival under physiologic conditions and are deregulated in many pathologies, e.g., human cancers. NcRNAs have been associated with cancer diagnosis, staging, treatment response, metastasis and survival and include distinct subtypes, e.g., long ncRNAs (lncRNAs) and microRNAs (miRNAs). LncRNAs have been linked to essential growth-promoting activities and their deregulation contributes to tumor cell survival. A prominent example is the *Hox* transcript antisense intergenic lncRNA, *HOTAIR*, that cooperates with the polycomb repressive complex to reprogram chromatin organization. *HOTAIR* expression is deregulated in a spectrum of cancers and *HOTAIR* expression correlates with patient survival. Here, we highlight emerging evidence that supports a role for lncRNAs in cancer with implications for the development of novel diagnostics and therapeutics.

## INTRODUCTION

Cancer remains a major challenge in modern medicine [1–6]. Despite improved diagnostics, an arsenal of FDA-approved anti-cancer drugs and targeted therapies, cancer remains a leading cause of death, a major economic burden and a severe limitation on patient quality-of-life. Moreover, in developed countries, cancer mortality has remained relatively constant for the past 35 years. Almost all of the ~500,000 annual cancer deaths in the U.S. are due, at least in part, to drug resistance [4–7]. Drug resistance, either *de novo* or acquired, still accounts for the majority of tumor relapses contributing to poor outcomes [6–10]. Drug combinations may prevent the emergence of resistance since a clone resistant to a single agent is more readily eradicated by multiple agents from different drug classes. Such a strategy has improved survival in certain cancers, e.g., acute lymphocytic leukemia, diffuse large B cell lymphoma, Hodgkin's lymphoma and germ cell tumors. However, drug resistance inevitably emerges in most cancer types to hinder patient response [11–21].

Numerous mechanisms may explain the molecular basis of drug resistance and treatment failure. Resistance results from genetic variations within the patient's tumor cell. Recently, the dormant state of cancer stem cells and the epithelial-mesenchymal transition have been linked to chemotherapeutic resistance [22–27]. While the design of cancer chemotherapy has become increasingly sophisticated, few agents universally prevent disease relapse. A common mechanism for the acquisition of resistance is expression of energy-dependent transporters that eject anticancer drugs from cells [28–30]. Induction of drug detoxification, reduced sensitivity to apoptotic signals, uncoupling of growth control and inactivation of cell death pathways all promote drug resistance in cancer cells. Recent evidence points to a relationship between the drug-resistant phenotype and epigenetic alterations within cancer cells [31–33]. Non-coding (ncRNAs) are major regulators of epigenetic, transcriptional and post-transcriptional gene expression. NcRNAs have also been reported to play a role in chemoresistance by impairing the response through cell cycle arrest, inhibition of apoptosis and enhanced DNA damage repair [34–37]. Here, we describe the rapidly emerging role of long ncRNAs (lncRNAs) in cancer drug resistance and highlight a prominent example, the lncRNA *HOTAIR,* to correlate lncRNA expression with metastasis, drug resistance and clinical outcome [38–40].

## NcRNAs AND CANCER

**Figure 1:**
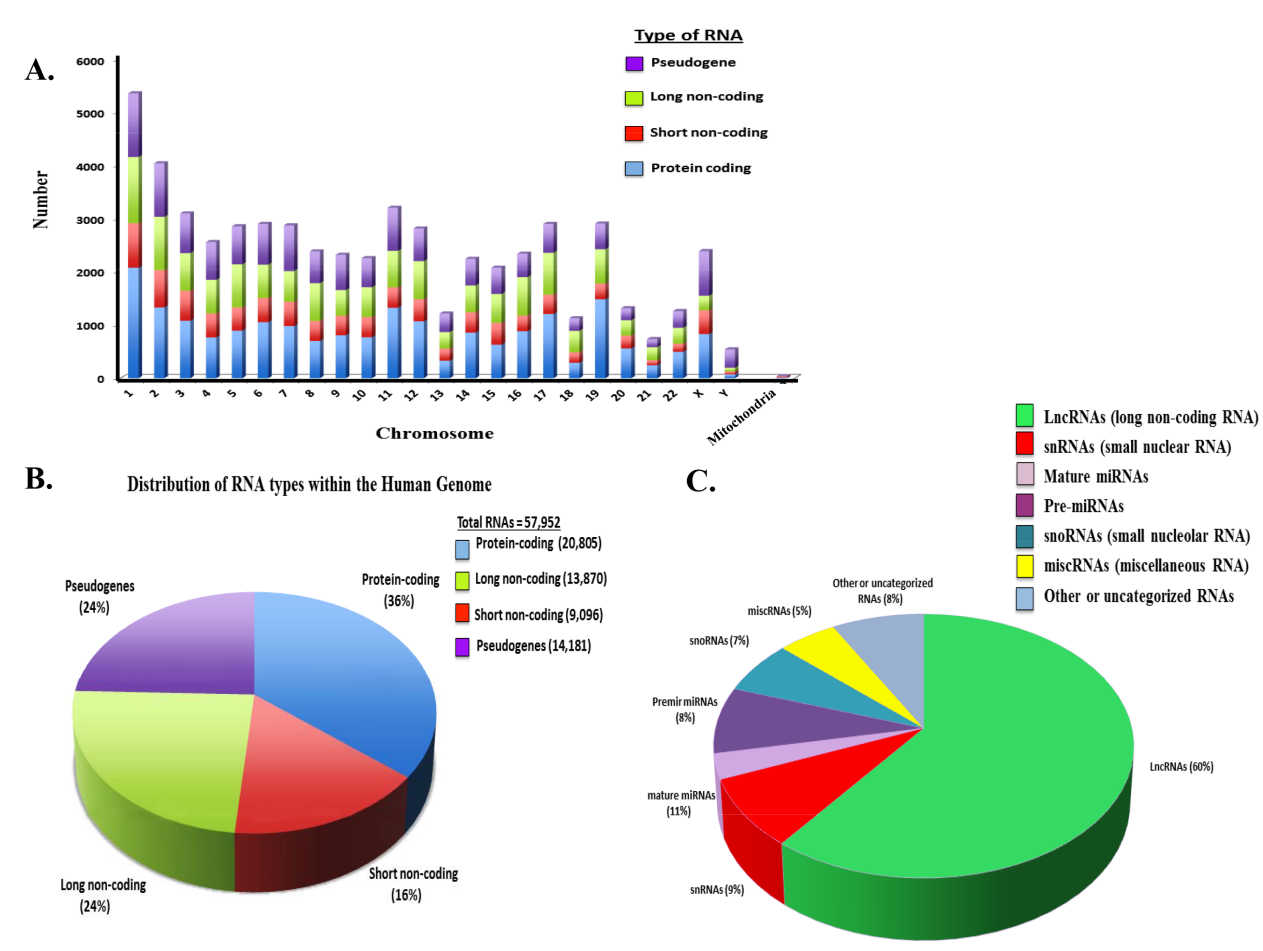
Distribution of Coding and Non-coding RNAs in the Human Genome **(A)** Distribution of different RNA types on the individual human chromosomes. Number of coding and ncRNAs is based upon http://useast.ensembl.org and Gencode19. It is noted that the most recent ensembl version indicates a total putative ~140,000 ncRNAs based upon whole genome sequencing. Numbers used here reflect on those RNAs that have been validated. **(B)** Distribution of RNA types within the human genome. **(C)** Distribution of non-coding RNA types within the human genome.

Studies have revealed that protein-coding and non-protein-coding genes are extensively transcribed within the human genome [41–44]. Protein-coding and non-protein-coding RNAs, such as long and short ncRNAs, are found on all human chromosomes (Figure 1A). This has led to the identification of novel classes of ncRNAs that regulate gene expression through diverse mechanisms. NcRNAs are deregulated or mutated in diseased cells from many human cancers [45–48]. NcRNAs represent a broad class of structurally and functionally distinct RNAs. These include long and short ncRNAs that represent a significant proportion (~60%) of the RNAs distributed throughout the human genome (Figure 1B) [49–52]. The distribution of lncRNAs and different short ncRNA types within the human genome is shown (Figure 1C).

For nearly four decades, mutation, amplification or deletions within protein-coding oncogenes and/or tumor suppressors was considered the primary cause of tumorigenesis [53–57]. This thinking has recently been revisited with the discovery of ncRNAs [58–60]. Thousands of genes that produce long or short ncRNA transcripts without any significant open reading frame have demonstrated that the complexity of cancer cell genetics is far greater than expected. These results led to a paradigm shift in addressing cancer biology and approaches in cancer treatment.

NcRNAs do not encode proteins but studies have documented their role during the steps of tumorigenesis and the development of therapeutic resistance [61–64]. NcRNAs are differentially expressed in a number of cancer types relative to the surrounding healthy (normal) tissue [65–67]. Importantly, many of the same ncRNAs appear to be commonly deregulated in multiple tumor types. While ncRNAs are newcomers in genome biology, these findings indicate that ncRNAs directly modulate key pathways that promote cancer growth. MiRNAs range from 18–25 nucleotides (nt) while lncRNAs extend up to >10,000nt. MiRNAs typically are excised from a 60 to 110nt hairpin precursor (pre-miRNA) that is transcribed from a larger primary transcript (pri-miRNA). MiRNAs bind target messenger RNA (mRNA) transcripts through sequence complementarity to either promote mRNA degradation or to prevent mRNA translation. While much is known about miRNAs, little exists regarding the biology and function of lncRNAs. While the precise functions of individual lncRNAs in cancer is only beginning to emerge, it is evident that ncRNAs are essential for tumorigenesis [68–71].

## LncRNA *HOTAIR* AND HUMAN CANCERS

LncRNAs are a heterogeneous group of RNAs that regulate expression at the epigenetic, transcriptional or post-transcriptional level [72–76]. LncRNAs bind specific gene clusters to not only prevent the binding of transcriptional activators but also recruit chromatin remodeling proteins as a molecular scaffold to silence gene expression. LncRNA can also interact with chromatin modifying proteins *in trans* to epigenetically silence genes at distant loci. LncRNA transcription may open the chromatin structure and permit access to the transcriptional machinery and enhance expression of neighboring protein-coding genes. Similarly, transcription of lncRNAs near protein-coding loci can repress transcription since the presence of the transcriptional machinery on the lncRNA gene locus physically prevents binding to protein-coding genes. The post-transcriptional roles of lncRNAs are more diverse. Since many lncRNAs are complementary to protein-coding genes, lncRNAs may function in mRNA splicing, editing, transport, translation and degradation. Although lncRNAs are best known for modulating transcription, their post-transcriptional influence on mRNA translation is emerging. Recently, *HOTAIR* was shown to be a post-translational inducer of ubiquitin-mediated proteolysis. *HOTAIR* associates with certain E3 ubiquitin ligases bearing RNA-binding domains, such as Dzip3 and Mex3b, as well as with their respective ubiquitination substrates, Ataxin-1 and Snurportin-1 [88]. *HOTAIR* facilitates the ubiquitination of Ataxin-1 by Dzip3 and Snurportin-1 by Mex3b and accelerates their degradation. These studies have uncovered a fascinating and unforeseen role for *HOTAIR* in protein degradation.

**Figure 2:**
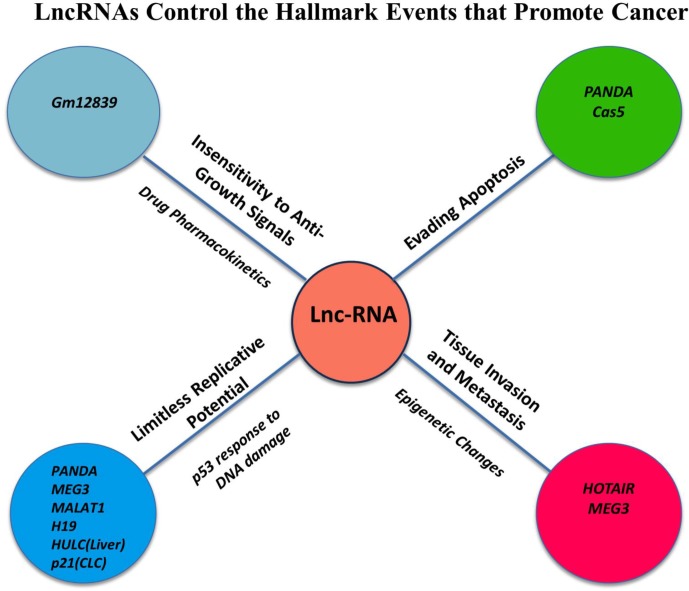
LncRNAs Control the Hallmark Events that Promote Cancer Initiation and Progression LncRNAs have been linked to the promotion of hallmark features of cancer cells including the insensitivity to anti-growth signals, evading apoptosis, limitless replicative potential and tissue invasion and metastasis.

The hallmarks of cancer comprise biological capabilities acquired during the multistep development of tumors [78, 79]. These features constitute an organizing principle for rationalizing the complexities of cancer and include sustaining proliferative signaling, evading growth suppressors, resisting cell death, enabling replicative immortality, inducing angiogenesis, and activating invasion and metastasis. Underlying these hallmarks is the common thread of genomic instability to generate the diversity that expedites tumor survival. Importantly, the results collectively indicate that lncRNAs regulate many genes that contribute to tumorigenesis and promote the hallmark features of cancer [Figure 2] [79–82].

### LncRNA expression and metastasis, drug resistance and overall survival

The *Hox* transcript antisense intergenic RNA *HOTAIR* is expressed from the developmental *HOXC* locus located on chromosome 12q13.13 and cooperates with the polycomb repressive complex PRC2 [83]. This 2.2kb spliced RNA transcript interacts with the PRC2 to modify chromatin and repress transcription of the *HOX* genes [84, 85]. Although less than 2% of a mammalian genome codes for protein, at least 50% is transcribed [85]. Recently it was shown that *HOTAIR* serves as a modular scaffold, assembling a molecular cargo of specific combinations of enzymes that are equipped to regulate target genes [86]. Further studies then demonstrated that *HOTAIR* reprograms chromatin organization and in breast cancer cells promotes metastatic potential [87].

### LncRNAs that correlate with metastasis

**Figure 3:**
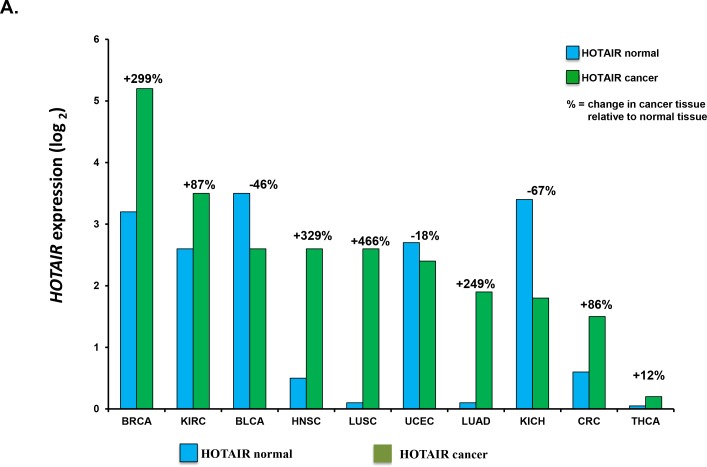
*HOTAIR* expression in cancer tissues and correlation with patient outcome **(A)** Correlation of *HOTAIR* expression in healthy vs. cancer tissue samples. *HOTAIR* expression was determined from indicated tissue samples and correlated to that in healthy (normal) tissue. Number of samples (n) = 5599. Data was obtained from the starBase Pan-Cancer Analysis Platform (http://starbase.sysu.edu.cn/panCancer.php) to explore Pan-Cancer Networks of lncRNAs, miRNAs, ceRNAs and RNA-binding proteins (RBPs) by mining clinical and expression profiles of 14 cancer types including tumor and normal samples from the Cancer Genome Atlas (TCGA) data portal. Cancer types used were from urothelial bladder (BLCA), breast (BRCA), colon and rectal adenocarcinoma (CRC), glioblastoma multiforme (GBM), head and neck squamous cell carcinoma (HNSC), chromophobe renal cell carcinoma (KIRH), clear cell kidney carcinoma (KIRC), acute myeloid leukemia (LAML), lung adenocarcinoma (LUAD), lung squamous cell carcinoma (LUSC), ovarian serous cystadenocarcinoma (OV), cutaneous melanoma (SKCM), papillary thyroid carcinoma (THCA) and uterine corpus endometrial carcinoma (UCEC). B. Correlation of *HOTAIR* expression with patient 5 year survival. Shown is the correlation of *HOTAIR* expression in tissue from non-small cell lung, colorectal, breast and cervical cancer patients.

*HOTAIR* expression has been investigated in tumor samples from many different cancer types. Healthy and cancer tissue was compared to demonstrate that *HOTAIR* is deregulated in many tumor types (Figure 3A). *HOTAIR* expression from patients diagnosed with stage IV colorectal cancer (CRC) and liver metastases was shown to correlate with a poor prognosis [89]. In addition, high expression of *HOTAIR* correlated with the presence of liver metastases. *MALAT1* (metastasis-associated lung adenocarcinoma transcript 1) is another lncRNA that has been associated with metastasis and poor prognosis for patients with NSCLC [90]. *MALAT1* resides on chromosome 11q13.1 which has been found to harbor chromosomal translocation breakpoints linked to cancer [91, 92]. Other *in vitro* studies, have implicated *MALAT1* in the regulation of the invasive potential of cancer cells, in cervical [93] and lung cancers [94].

### LncRNAs that correlate with therapeutic resistance

The lncRNA cancer upregulated drug resistance gene (*CUDR*) is a 2.2kb RNA transcript that antagonizes the apoptotic effect of cisplatin in bladder cancer cells [95]. *CUDR* expression correlates with tumorigenesis as well as cellular growth [96]. *CUDR* has been shown to render resistance to doxorubicin and etoposide, commonly used for squamous cell cancer. The growth arrest-specific 5 lncRNA *Gas5* contributes to glucocorticoid resistance [97]. *Gas5* exerts its effects by assuming a secondary structure that is similar to a sequence within the steroid-responsive gene promoter. The result is that *Gas5* competitively inhibits steroid receptor binding to the steroid-responsive region of the promoter to prevent transcriptional activation. A role as a tumor suppressor in renal cell carcinoma has also recently been reported [98]. *PANDA* is a lncRNA increased in a subset of breast cancer cells that contributes to anthracycline resistance, a crucial component of breast cancer chemotherapy [99]. *PANDA* acts by interacting with the nuclear transcription factor NF-YA to decrease the apoptotic effect. *PANDA*-depleted fibroblasts have been shown to display increased sensitivity to chemotherapy [100]. A large intergenic ncRNA induced by p53 mediates global gene repression and many p53-regulated lncRNAs are induced in response to DNA damage and promote chemoresistance. *H19* knockdown by transfection with antisense *H19* oligonucleotides suppresses the multi-drug resistance gene and promotes doxorubicin sensitivity [101, 102]. Finally, *HULC* is a 1.6 kb lncRNA that may act as an endogenous sponge to reduce miRNA levels and inhibit their functional activity [103, 104].

### LncRNAs in metabolic inactivation of anti-cancer drugs

The *Drosophila sechellia* lncRNA *Gm12839* is located ~40kb downstream of a cytochrome P450 system gene [105]. *Gm12839* exhibits a reverse expression pattern with the *P450* genes to suggest an inhibitory effect on the cytochrome P450 drug metabolism and may be crucial in the inactivation of chemotherapeutics.

### *Hotair* expression correlates with overall survival

*HOTAIR* expression in tissue from patients diagnosed with either non-small cell lung, colorectal, breast or cervical cancers was the correlated with five year overall survival (Figure 3b.) We found that patients with increased *HOTAIR* expression had a worse survival in all four cancer types studied. These and related studies may soon lead to the development of lncRNA signatures that classify patients into high and low-risk groups with significantly different survival rates. The prognostic value of lncRNA signatures needs to be confirmed in independent data sets but the identification of prognostic lncRNAs indicates their potential.

## *HOTAIR R*EGULATION OF CHROMATIN REMODELING

**Figure 4:**
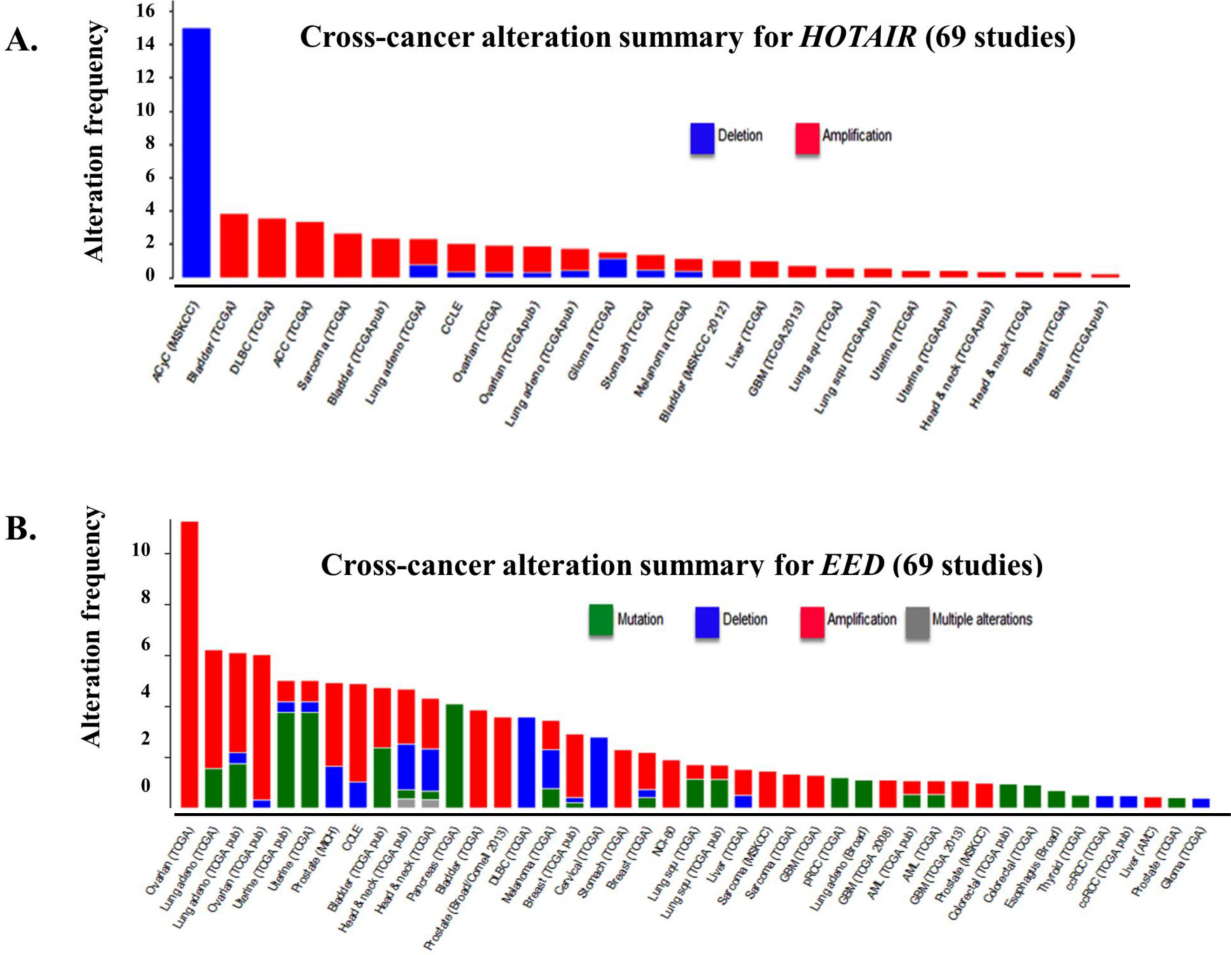
Frequency of *HOTAIR* alteration and putative *HOTAIR*-controlled genes across cancer types **(A)** Cross-cancer alteration summary for *HOTAIR* from 69 independent studies to correlate *HOTAIR* expression with alteration frequency. Potential alterations included mutation, deletion, amplification or multiple alterations. Data was obtained from the cBioportal for cancer genomics (http://www.cbioportal.org/public-portal/, Memorial Sloan-Kettering Cancer Center, NY, NY). **(B)** Shown is the correlation between the alteration of *EED1*, a putative target of *HOTAIR*, across different cancer types. Potential alterations included mutation, deletion, amplification or multiple alterations. Data was obtained from the cBioportal for cancer genomics (http://www.cbioportal.org/public-portal/, Memorial Sloan-Kettering Cancer Center, NY, NY).

In a subset of 32 CRC specimens, gene set enrichment analysis revealed a correlation between *HOTAIR* expression and the PRC2 complex members, namely SUZ12, EZH2 and H3K27me3 [106]. *HOTAIR* may be associated with the genome-wide reprogramming of PRC2 in a broad number of tumor types. Approximately 20% of the identified lncRNAs regulate the transcriptional activity of protein-coding genes by guiding the histone methyltransferase PRC2 to specific genomic loci. Chemotherapy has been shown to induce epigenetic changes which contribute to the emergence of drug resistance [107]. We correlated the cross-cancer alteration of *HOTAIR* through either mutation, deletion, amplification with a broad number of cancer types (Figure 4A) [108]. *HOTAIR* was significantly deregulated in a significant percentage of patients from the different cancer types examined.

*HOTAIR* contains 6,232bp and encodes a 2.2kb lncRNA located within a HOXC gene cluster. It is shuttled from chromosome 12 to chromosome 2 by Suz-Twelve. The 5' end of *HOTAIR* interacts with PRC2 and as a result regulates chromatin state. The 3' end of *HOTAIR* interacts with the histone demethylase LSD1. PRC2 consists of four subunits: Suz12, *Eed, Ezh1* or *Ezh2* (SET domain with histone methyltransferase activity and *RbAp48* (histone-binding domain). *HOTAIR* may contribute to the regulation and functioning of the PRC2 subunits. In addition to *HOTAIR*, a correlation between alterations in *EED1* was also seen across the different cancer types [Figure 4B]. Future studies will further define the role of *HOTAIR* on PRC2, chromatin remodeling and individual components, e.g., *EED1*, of PRC2.

## LncRNA THERAGNOSTICS

Theragnostics, a portmanteau of therapeutics and diagnostics, describes a system to customize healthcare using molecular and genetic tools for treatment decisions tailored to the individual patient. LncRNAs are therefore perfectly tailored as cancer theragnostics to design personalized medicines based upon molecular features of each tumor [109, 110]. Novel cancer diagnostics, prognostics and anti-cancer strategies based upon ncRNA biology are rapidly emerging. For example, lncRNAs isolated from tumor cells or circulating within the bloodstream may provide for readily-available, inexpensive and stable blood-borne diagnostics to more readily detect cancers and cancer subtypes. Interference of RNA expression to treat cancer has recently gained momentum as a treatment modality since growth-promoting lncRNAs may be inactivated through antisense technologies. LncRNAs may also function as mRNA or miRNA sponges to inactivate growth-promoting, pro-tumorigenic signaling pathway. Therefore, synthetically-engineered lncRNAs may be employed through replacement therapy to inhibit tumor cells [111]. Cancer cells have increased the protein synthesizing machinery as well as enhanced ncRNA transcription rates making RNA interference an attractive strategy. NcRNAs offer potential as therapeutic targets is exemplified by Onconase, a non-specific ribonuclease which neutralizes tRNA, rRNA, mRNAs and ncRNAs. Onconase, currently in phase II trials, induces a G1 block in the cell cycle and increased apoptosis [112]. Delivery vehicles, e.g., liposomal membranes, carry growth-inhibitory ncRNAs into tumor cells as another lncRNA-based treatment strategy. MRX34 is a ncRNA-based therapeutic that targets miR-34 is being evaluated in clinical trials for hepatocellular cancers [113, 114]. The absence of nucleases within the cerebral spinal fluid (CSF) has made intrathecal injection of RNA-based agents an attractive approach to treat neurodegenerative diseases. Primary brain tumors and brain metastases may similarly be targeted. Nanoparticles that deliver RNA inhibitors are also emerging [115–117]. Dextran nanoparticles can deliver chemotherapy to the nucleus and may be used to attach cytotoxic agents to lncRNAs. The plasmid DTA-H19 was designed by express a diphtheria toxin subunit controlled under the *H19* promoter [118, 119]. Intratumoral injection of this compound can lead to a H19-dependent activation of diphtheria toxin within the tumor. Another emerging modality is to increase tumor suppressor activity by neutralizing the inhibitory of effect of lncRNAs through antisense oligonucleotides. These compounds do not need any delivery vehicle and can be injected subcutaneously and are directly taken up by cells. They can cross endosomal membranes, enter the nucleus and inhibit PRC2 interaction with lncRNAs. These compounds may also cross the blood brain barrier without the need of a lipid carrier.

## CONCLUSIONS

LncRNAs are rapidly being recognized as important regulators of gene expression in cancer. The evolutionary conservation, diversity and complexity of lncRNAs indicates that they exert significant regulatory control on cell growth. While understanding the mechanistic role of lncRNA in cancer is still early, general principles are emerging. LncRNAs, e.g., *HOTAIR*, are deregulated in many cancer types, affect signaling pathways that promote cancer and modulate gene expression. Future studies will identify similar lncRNAs leading to signatures to better diagnose cancers. LncRNAs should allow for better stratification of cancer subtypes to correlate lncRNA expression with therapeutic response, drug resistance and overall survival. Similar to miRNA expression profiling in multiple myeloma and related B cell malignancies, lncRNAs profiling should lead to the identification of genetic subtypes and their association with treatment response and survival [120–122]. Finally, the structural and functional novelty of lncRNAs offers promise as anticancer therapeutics that may avoid the emergence of drug resistance commonly seen with the currently used agents. Studies to better understand the molecular mechanisms of lncRNAs in cancer offers promise for the development of more effective cancer therapies.
